# 2536. Comparing the activity of broad-spectrum beta-lactams in combination with aminoglycosides and aztreonam/avibactam against 8 VIM-producing CRE

**DOI:** 10.1093/ofid/ofad500.2153

**Published:** 2023-11-27

**Authors:** Justin A Clark, David Burgess

**Affiliations:** University of Kentucky College of Pharmacy, Lexington, Kentucky; UK HealthCare, Lexington, KY

## Abstract

**Background:**

Treatment of infections caused by CRE often involves beta-lactam-containing combination therapy, either with a beta-lactamase inhibitor or an agent from a different antimicrobial class. We sought to investigate the activity of several beta-lactam combinations against 8 VIM-producing CRE cultured from patients at our academic medical center using a time-kill assay.

**Methods:**

Broth microdilution was utilized to obtain the MICs. TK was performed in duplicate with samples taken 0, 4, 8, and 24 hours after initial inoculation. During each time point, samples were automatically spiral plated onto Mueller Hinton agar plates and incubated for 16-24 hours at 37 ^o^C before colonies were enumerated to estimate bacterial density (CFU/mL). Antimicrobials tested included meropenem 16 μg/mL, cefepime 32 μg/mL, piperacillin/tazobactam 64/4 μg/mL, amikacin 4 μg/mL, plazomicin 4 μg/mL, and aztreonam/avibactam 32/4 μg/mL. Average bacterial density reduction after 24 hours was the outcome of interest.

**Results:**

Meropenem and plazomicin alone achieved bactericidal or bacteriostatic activity in at least a single time-kill experiment for all isolates except for meropenem against one isolate. Aztreonam/avibactam and meropenem with either amikacin or plazomicin achieved bactericidal activity by 4 hours which sustained until 24 hours except for a single isolate in which meropenem/plazomicin only achieved bacteriostatic activity. These combinations were the most active with average bacterial density decreases of 3.86, 3.89, and 3.64 log_10_reductions, respectively. Cefepime or piperacillin/tazobactam combined with plazomicin minimally improved the average bacterial inocula reduction compared to plazomicin monotherapy. When combined with amikacin, cefepime and piperacillin/tazobactam achieved inconsistent bactericidal activity after 24 hours, though cefepime on average led to a bacterial reduction > 2 log_10_ CFU/mL lower than piperacillin/tazobactam.

**Conclusion:**

Against 8 clinical VIM-producing *K. pneumoniae* and *E. cloacae*, beta-lactam combination therapy achieved rapid and sustained bactericidal activity. Further investigation is necessary to rationally guide the use of these combination therapies against CRE.

**Disclosures:**

**All Authors**: No reported disclosures

